# Intraoral vs. extraoral bitewing radiography for approximal caries detection: A multi-observer ex vivo ROC study using thin-section microscopy as gold standard

**DOI:** 10.1007/s00784-025-06511-1

**Published:** 2025-09-26

**Authors:** Julia Caroline Quintus, Ralf Kurt Willy Schulze

**Affiliations:** 1 Department of Orthodontics, University Medical Center of the Johannes Gutenberg University Mainz, Mainz, Germany; 2 Present Address: Oral Diagnostic Sciences, School of Dental Medicine, University of Bern, Bern, Switzerland

**Keywords:** Interproximal caries detection, Bitewing radiography, Extraoral bitewing radiography, Receiver operating characteristic (ROC) analysis

## Abstract

**Objectives:**

This ex vivo study aimed to compare the accuracy in detection of interproximal natural carious lesions between intraoral (iBWR) and extraoral bitewing radiographs (eBWR) using a multi-observer design and a rigorous gold standard.

**Materials and methods:**

Eighty extracted teeth (40 premolars, 40 molars) were arranged in anatomical sequence within a simulated jaw composed of PMMA and modified gypsum, with an emphasis on creating natural interproximal contacts. Approximately 50% of the teeth exhibited enamel caries, while the remaining 50% were caries-free. Image acquisition was performed using a custom-designed PMMA phantom. iBWR were obtained with a CMOS intraoral sensor (XIOS XG Supreme, Sirona Dental Systems, Bensheim, Germany), and eBWR with a digital panoramic device (Orthophos SL 3D, Dentsply Sirona, Bensheim, Germany).

Twenty-seven licensed dentists assessed caries presence and depth on 120 approximal surfaces (each surface assessed twice using both modalities) using a 5-point confidence scale and a 4-point lesion depth scale. Observers were blinded to the true caries status, which was determined through histological serial sectioning and brightfield microscopy. Diagnostic accuracy was evaluated via ROC analysis, with Youden’s index used to calculate sensitivity, specificity, predictive values, and likelihood ratios. Statistical analyses were conducted at a significance level of α = 0.05.

**Results:**

Overall accuracy was higher for iBWR (Az_pooled_ = 0.58) than for eBWR (Az_pooled_ = 0.54). Both intra-rater (test-retest, eBWR $\:\rho\:$_spearman_ = 0.44, iBWR $\:\rho\:$_spearman_ = 0.48) as well as inter-rater reliability (mean ICC eBWR = 0.19, iBWR = 0.27) were low. For enamel caries detection, iBWR outperformed eBWR in terms of specificity and positive predictive values, while eBWR in the first reading round achieved significantly higher sensitivity.

**Conclusions:**

Overall, our multi-observer ex vivo study using microscopy as ground truth revealed higher diagnostic accuracy for intraoral bitewing radiography as compared to its extraoral counterpart.

**Clinical Relevance:**

Our results from a highly standardized study using a rigorous gold standard support the assumption that intraoral bitewing radiography still represents the radiographic state-of-the-art in interproximal caries detection. For minute enamel, diagnostic accuracy of both methods is just above random guessing.

## Introduction

Despite their long-term existence, intraoral bitewing radiographs (iBWR) still are the standard of care particularly for diagnosing interproximal caries [1, 2]. Dental caries is a prevalent oral disease, with an estimated (33.6%) of caries in of permanent teeth affecting 294 million people in the European Region [3]. For the US, the prevalence of total dental caries (untreated and treated) in primary or permanent teeth among youth aged 2–19 years was 45.8% in 2015 to 2016 [4].

These figures indicate that simple and readily available techniques to detect carious lesions are required. BWR taken with typical intraoral radiographic tubes and equipment provide a solution. Compared with apical projections, iBWR were shown to provide significantly better sensitivity for all levels of caries progression (iBWR: 94.5 for dentin caries, 90.43 to 82.7 for enamel caries, periapical: 69.7 for dentin caries, 39.01 to 56.2 for enamel caries) [5]. However, interestingly the authors observed no difference regarding specificity [5]. One important factor in favor of BWR may be the relatively standardized projection geometry with the image receptor placed parallel to the long axes of the crowns. However, the horizontal projection angle is a critical point, and thus longer receptors (such as size 3) are not recommended since they increase proximal surface overlapping in their periphery [6]. Intraoral BWR may induce patient discomfort and the possibility of cross-contamination.

An extraoral solution termed “extraoral bitewings” (eBWR), based on a specialized panoramic image acquisition technique, was introduced around 2012 [7]. Since then, a variety of authors have investigated the technique with inconsistent observations. While Kamburoglu and colleagues using film as detector for iBWR found superior performance of iBWR compared to extraoral bitewing and panoramic radiography in diagnosing proximal caries of premolar and molar teeth ex vivo [7], Terry and colleagues observed no significant difference in posterior proximal surface caries detection between the modalities [8]. Terry et al. used storage phosphor plates as iBWR-receptors and a “gold standard” established by two experts on the basis of the iBWR. The latter is known to provide a weak standard since the method under investigation is identical to the method used to define the gold standard [9]. For artificially induced “caries lesions”, Abu El-Ela and colleagues in 2016 observed no significant differences between iBWR (phosphor plates and CMOS-sensor) and eBWR [10]. This ambiguity in the different studies may be due to design differences, yet also due to sample size.

To further investigate this interesting topic, this study was designed. Other than the studies described above, our investigation was based on (i) multiple observers and (ii) histological sections in combination with microscopy to achieve a most accurate gold standard. A multi-observers design design was used to reduce bias due to the well-known inter-observer variance in radiological diagnosis [11, 12].

Aim of this study was to, using a multi-observer set-up, investigate probable differences between iBWR (acquired with a digital intraoral detector) and eBWR (acquired with a state-of-the-art digital panoramic device) with respect to detection of interproximal caries. A rigorous accuracy analysis was conducted by comparing to a best-possible golden standard, i.e. histological sections of the teeth. Our null hypothesis was that the diagnostic accuracy for interproximal caries detection does not differ between the two modalities.

## Materials and methods

As phantom study, no ethical approval was required. The teeth for the phantom were taken from regular extractions under the consent of the patients for secondary scientific use.

### Phantom

**Fig. 1:**
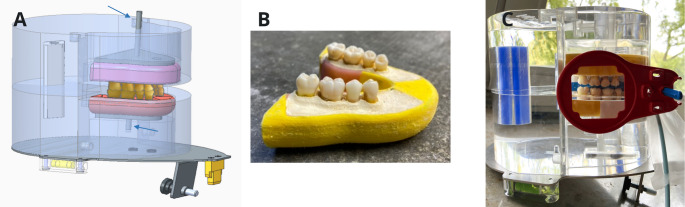
**A**: Drawing of the PMMA-phantom containing the tooth-bearing maxillary and mandibular units (**B**) made of superhard stone. The yellow insert at the bottom of the phantom fits into the respective trough in the panoramic machine. **C** displays the entire phantom with tooth-bearing unit plus the radiographic holding device for iBWR in place

The test phantom, developed with the support of Sirona Dental Systems GmbH, consisted of a transparent polymethylmethacrylate (PMMA) head phantom (Fig. 1A and C) serving as a scatter body, with scaffolds for inserting self-fabricated, tooth-bearing maxillary and mandibular units. The acrylic phantom had a thickness of 2.5 to 3 cm in the simulated cheek region. A large opening allowed external routing of the intraoral sensor cable and holder.

PMMA inserts with 2-mm-thick walls, precisely designed to fit into the phantom scaffolds in the upper and lower jaw regions, served as the tooth-containing units for positioning in the phantom. Preliminary tests evaluated substances and mixtures to replicate the radiographic appearance of human maxillary and mandibular bone.

A total of 80 teeth (40 premolars and 40 molars) from regular extractions were arranged in their natural anatomical sequence in the jaws. Approximately 50% of the teeth had enamel caries (visually identified as white/brown discolorations or small cavitations), while the remaining 50% were visually assessed as caries-free. Teeth with restorations, extensive caries, or prior endodontic treatments were excluded.

The teeth were initially fixed in the PMMA inserts using hot wax to mimic natural dentition, with particular attention to creating “natural” interproximal contacts between the teeth. A silicone impression (Dentalsilikon Monosil Twin 90, HLW Dentalinstruments GmbH, Wernberg-Köblitz, Germany) was used to stabilize the position of the teeth, enabling the removal of the wax and its replacement with the final embedding medium. To simulate a periodontal ligament gap, the tooth roots were briefly immersed in hot wax, forming a thin wax layer.

Preliminary tests demonstrated that a mixture of 100 g superhard stone (HS-Superhartgips gelbbraun, Henry Schein Dental Deutschland GmbH, Langen, Germany) and 22 ml water containing a calcium effervescent tablet produced a realistic bone-like radiographic appearance. This mixture was poured into the PMMA inserts, and the silicone impression containing the arranged teeth was placed into the fluent stone. After setting, the models (Fig. 1B) were consecutively positioned in the PMMA phantom for radiographic exposure (Fig. 1C). A total of five upper and five lower jaw models, i.e. five patient phantoms, were produced from a total of 80 teeth.

### Image acquisition

IBWR were acquired using a complementary metal-oxide-semiconductor (CMOS) intraoral sensor (XIOS XG Supreme, Sirona Dental Systems, Bensheim, Germany) with a square pixel size of 0.015 mm and an active area of 36 mm × 25.6 mm (1200 × 868 pixel). The PMMA-inserts containing the teeth were sequentially positioned in the phantom. iBWR were produced at 60 kV and 7 mA using a typical aiming system (Aimright, Sirona Dental Systems, Bensheim, Germany). Due to the holder system, the teeth of the upper and lower jaw models were realistically not in occlusal contact, and a source-to-receptor distance of approximately 268 mm was maintained.

EBWR were exposed using a digital panoramic device (Orthophos SL 3D, Dentsply Sirona, Bensheim, Germany). The PMMA phantom, containing the jaw inserts, was positioned in the panoramic device using a custom-designed metal mount. To simulate the spacing that typically occurs when patients bite on an anterior bite block, pink modeling wax (1 × 3 cm; Henry Schein Dental Deutschland GmbH, Langen, Germany) was bilaterally placed as a 1.5 cm spacer between the teeth. The “BW 1” program, specifically designed for lateral eBWR, was used for imaging. According to the manufacturer, this program applies a slightly wider primary slit collimation compared to standard panoramic radiography and uses a modified motion trajectory for the X-ray source and detector. eBWR were acquired at 60 kV and 8 mA with an exposure time of 8.8 s, producing images with a pixel size of 0.1 mm (1708 × 956 pixel).

**Fig. 2:**
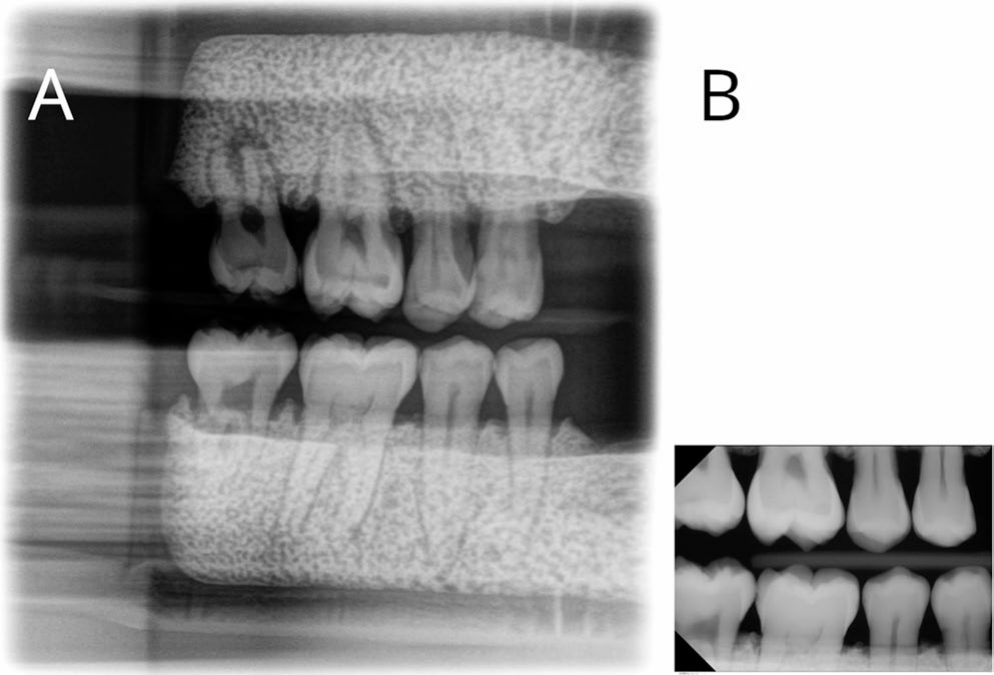
Exemplary eBWR (**A**) and iBWR (**B**) as obtained from the phantom. Note that the size of the two images in this Figure does not correctly reflect the actual 1:1 display that was used for image assessment

Figure 2A and B display an extraoral and an intraoral bitewing radiograph of the same test teeth examined in this study.

### Image evaluation/viewing sessions

Raters were selected from the dentists available at the Dental Hospital of the University Medical Center of the Johannes Gutenberg-University of Mainz. We aimed to include a sufficiently large rater-sample comprising of different sub-specialties. Twenty-seven licensed dentists (mean radiographic interpretation experience: 6.2 years; range: 2.7–17.0 years) participated. Of these, 25 worked in departments of the Dental Hospital (five in Periodontology and Conservative Dentistry, 12 in Prosthodontics and Dental Materials, and eight in Oral and Maxillofacial Surgery), while two were not employed at the time of the viewing sessions.

Image evaluations were conducted on a 19-inch LED display (Model B19-7 LED, Fujitsu Minato, Tokyo, Japan [1280 × 1024 pixel, luminance = 250 cd/m²]) in a quiet, darkened room. Quality checks were performed on evaluation days according to German DIN 6868 − 157 standards using the TG18-QC test pattern [13]. Images were displayed in 1:1 format, meaning that one sensor pixel is represented in one monitor pixel.

Observers received standardized instructions, including graphical explanations and example radiographs (not part of the study sample) to illustrate caries depth classification. Each observer, blinded to the true caries status, evaluated 20 radiographs (10 iBWR, 10 eBWR) corresponding to 80 teeth and 120 proximal surfaces. Radiographs were presented in a randomized order using proprietary software (Sidexis XG, Dentsply Sirona, Bensheim, Germany), routinely used at the University Medical Center Mainz. Observers could freely adjust image size, contrast, and brightness without time constraints. Raters were asked to evaluate the interproximal surfaces between the two premolars, between the premolar and first molar, and between the two molars, coronal to the cemento-enamel junction [14]. Possible carious lesions at the occlusal surfaces or in the cervical roots were not considered. Caries presence was rated on a 5-point confidence scale, ranging from 1 (“approximal caries definitely not present”) to 5 (“approximal caries definitely present”) [15]. Lesion depth was classified on a 4-point scale: 1 = outer enamel, 2 = inner enamel, 3 = outer dentin, and 4 = inner dentin.

Per session, a total of 240 approximal surfaces were evaluated (120 surfaces from 80 teeth, each assessed twice using two radiographic techniques).

To examine reliability, 24 observers repeated the evaluation after at least 30 days, while three raters were unavailable due to scheduling conflicts.

### Histological assessment

To validate the caries status, 78 study teeth (two teeth were lost during histological processing) were embedded in an MMA-based plastic embedding system (Technovit 9100, Kulzer Technik GmbH, Wehrheim, Germany) following the recommendations of Willbold and Witte (2010) [16]. The teeth were serially sectioned perpendicular to the central axis in a mesiodistal direction using the Exakt-Trennschleifsystem (EXAKT Advanced Technologies GmbH, Norderstedt, Germany) based on the sawing and grinding technique described by Donath and Breuner in 1982 [17]. Depending on tooth diameter, four to 12 thin sections were prepared per tooth.

Sections were analyzed using brightfield microscopy (Biorevo BZ-9000 microscope, Keyence, Osaka, Japan) at 2× magnification. Caries depth on proximal surfaces above the cemento-enamel junction was assessed by one of the study’s authors. Based on the definition by Hintze and Wenzel in 2003, a “whitish, opaque to brown/dark discoloration” was classified as a carious lesion (189. Lesions were categorized into five classes: 0 = no lesion, 1 = lesion in outer enamel, 2 = lesion in inner enamel, 3 = lesion in outer dentin, and 4 = lesion in inner dentin [18].

### Statistical analysis

Intrarater reliability was assessed by means of the Spearman’s rank correlation coefficient, while inter-rater reliability was assessed using the intraclass correlation coefficient (ICC) according to Shrout and Fleiss [19].

To assess the diagnostic accuracy of both imaging techniques, observers’ evaluations were compared to the histological gold standard through receiver operating characteristic (ROC) analysis. All statistical analyses were performed using R 4.3.2 [20] with RStudio 4.0.735 [21]. ROC analysis was conducted using the pROC package (v1.18.5 [22]). To calculate sensitivity, specificity, positive and negative predictive values, as well as positive and negative likelihood ratios, the threshold with the highest Youden index was selected from the ROC analyses. A significance level of α = 0.05 was applied for all statistical calculations. For assessment of relevant factors (imaging method, lesion depth) on performance differences an analysis of variance was computed.

## Results

### Histological evaluation

Of the 117 examined proximal surfaces, 58 (49.57%) showed carious lesions: 19 in outer enamel (D1, 32.76%), 23 in inner enamel (D2, 39.66%), 15 in outer dentin (D3, 25.86%), and one in inner dentin (D4, 1.72%).

### Intra- and interrater reliability

Intrarater reliability, assessed using Spearman’s rank correlation coefficient, showed $\:\rho\:$_mean_ = 0.44 (SD = 0.16, range $\:\rho\:$ = 0.18–0.70) for extraoral and $\:\rho\:$_mean_ = 0.48 (SD = 0.16, range $\:\rho\:$ = 0.12–0.90) for intraoral bitewing radiographs. Although the mean intrarater reliability coefficients were significantly different from zero, they indicated only low reliability according to Koo and Li’s classification [20]. We found no significant difference between the two modalities (z = −0.16, *p* = 0.87).

Interrater reliability was assessed using the intraclass correlation coefficient [ICC [1, 2] as described by Shrout and Fleiss [21]. For extraoral radiographs, ICC [1, 2] was 0.19 (*p* < 0.01, CI = [0.14; 0.24]) at both sessions. For intraoral radiographs, ICC [1, 2] was 0.28 (*p* < 0.01, CI = [0.23; 0.35]) at the first session and 0.25 (*p* < 0.01, CI = [0.20; 0.32]) at the second. Although the mean interrater reliability coefficients were significantly different from zero, they also indicated only low reliability according to Koo and Li’s classification [23].

### Diagnostic accuracy

**Fig. 3:**
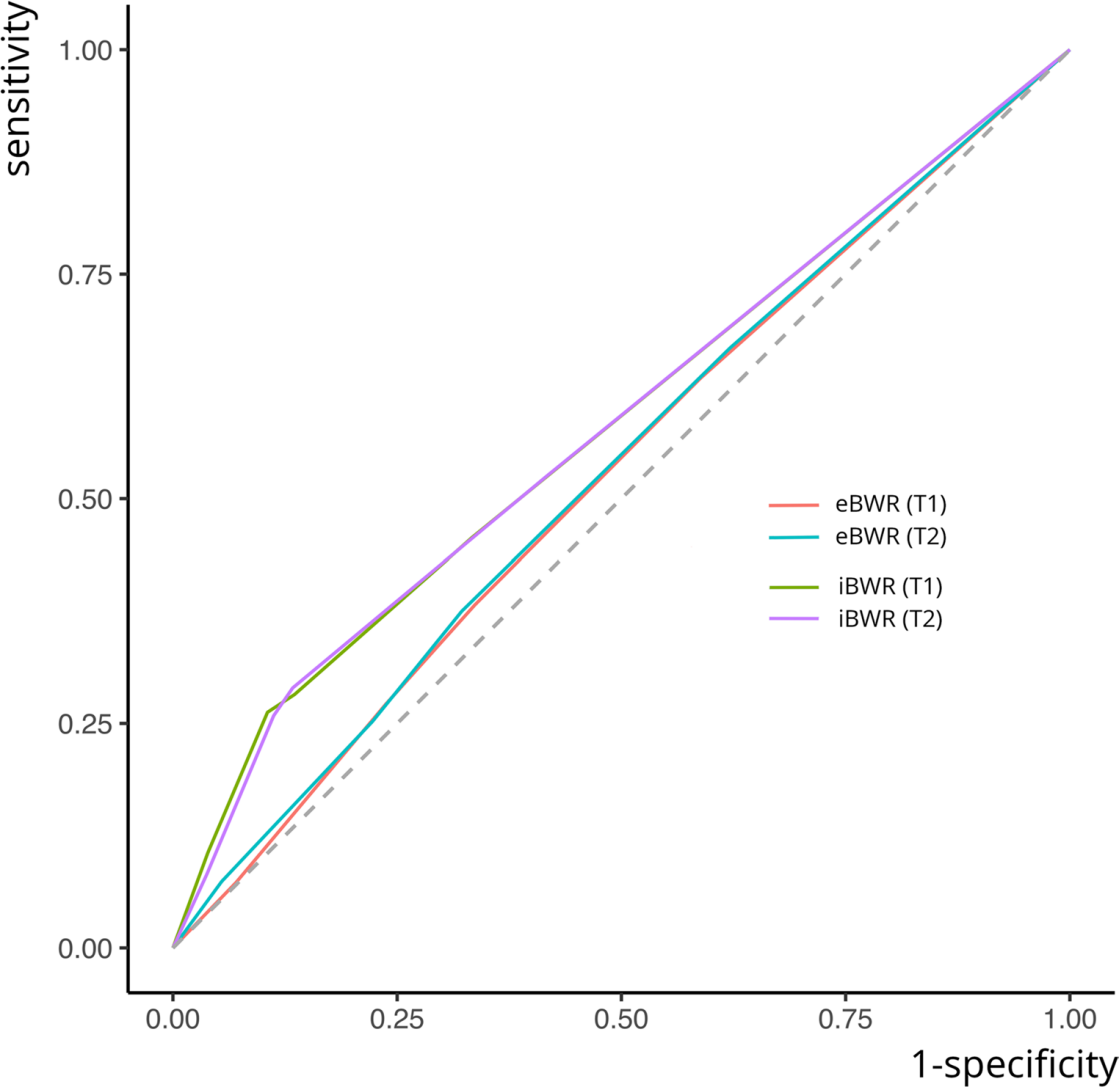
ROC-curves for accuracy analysis pooled over all raters yet separated for the two observations. Both ROC curves for extraoral bitewing radiographs are positioned just above the 45° diagonal at both test sessions, indicating low diagnostic accuracy. Clearly, both ROC curves for intraoral bitewing radiographs demonstrate a greater distance from the 45° diagonal, reflecting significantly yet only slightly higher diagnostic accuracy

As shown in Table 1, the AUC values for diagnostic accuracy were 0.527 (T1) and 0.545 (T2) for extraoral bitewing radiographs, and 0.580 (T1) and 0.586 (T2) for intraoral bitewing radiographs, indicating a poor diagnostic performance for both imaging techniques [24]. However, AUC values for intraoral bitewing radiographs were significantly higher than those for extraoral bitewing radiographs (Fig. 3), as determined by t-tests (t [23]T1 = 5.369, *p* < 0.01, d = 1.01; t [23]T2 = 4.332, *p* < 0.01, d = 0.88), with large effect size at T1 and medium effect size at T2 [25].

**Table 1:** Comparison of diagnostic metrics between extraoral (highlighted in light gray) and intraoral bitewing radiographs

Method	Observation No (T)	Parameter [mean ± SD; 95%-CI]
		**AUC**
eBWR	1 (T1)	0.527 ± 0.045; 0.440–0.638
	2 (T2)	0.545 ± 0.042; 0.463–0.654
iBWR	1 (T1)	0.580 ± 0.050; 0.511–0.699
	2 (T2)	0.586 ± 0.050; 0.476–0.683
		**sensitivity**
eBWR	1 (T1)	0.504 ± 0.248; 0.103–0.983
	2 (T2)	0.439 ± 0.218; 0.069–0.845
iBWR	1 (T1)	0.309 ± 0.140; 0.103–0.569
	2 (T2)	0.360 ± 0.145, 0.069–0.586
		**specificity**
eBWR	1 (T1)	0.586 ± 0.237; 0.085–0.915
	2 (T2)	0.666 ± 0.203; 0.288–1.000
iBWR	1 (T1)	0.870 ± 0.096; 0.576–0.983
	2 (T2)	0.823 ± 0.124; 0.559–0.983
		**PPV**
eBWR	1 (T1)	0.553 ± 0.036; 0.509–0.633
	2 (T2)	0.597 ± 0.109; 0.510–1.000
iBWR	1 (T1)	0.723 ± 0.098, 0.569–0.909
	2 (T2)	0.695 ± 0.105; 0.553–0.947
		**NPV**
eBWR	1 (T1)	0.569 ± 0.073; 0.509–0.833
	2 (T2)	0.556 ± 0.039; 0.509–0.654
iBWR	1 (T1)	0.565 ± 0.036; 0.520–0.658
	2 (T2)	0.570 ± 0.034; 0.514–0.648

*SD* standard deviation; *95%-CI *95%-Confidence interval, *auc *area under the Curve,* ppv *positive predictive Value,* npv *negative predictive Value,* LR + *Positive likelihood Ratio,* LR- *Negative likelihood ratio

Sensitivity values were however higher for extraoral than for intraoral bitewing radiographs. At T1, this difference was significant (W_T1_ = 142, *p* < 0.01, *r* = 0.62), corresponding to a medium effect size, while at T2, no significant difference appeared (W_T2_ = 125, *p* = 0.09).

Specificity values were in turn significantly higher for intraoral bitewing radiographs at both test sessions (*W*_T1_ = 168, *p* < 0.01, *r* = 0.73; *W*_T2_ = 172, *p* < 0.01, *r* = 0.62), corresponding to a medium effect size.

Positive predictive values and positive likelihood ratios were also significantly higher for intraoral bitewing radiographs at both sessions (all *W*s $\:\ge\:$ 170, all *p*s < 0.01, all rs $\:\ge\:$ 0.88) with medium to large effect sizes. In contrast, no significant differences were found between the two imaging techniques for negative predictive values and negative likelihood ratios (all *W*s $\:\ge\:$ 89, all *p*s $\:\ge\:$ 0.10).

Table 1 presents the diagnostic metrics for assessing the diagnostic accuracy of extraoral and intraoral bitewing radiographs, separated by both test sessions.

#### Enamel versus dentin caries

To examine whether the diagnostic accuracy of both techniques was influenced by caries depth, a follow-up analysis differentiated between enamel and dentin caries. Of the 117 approximal surfaces, 42 surfaces (35.90%) showed carious lesions confined to enamel, and 16 surfaces (13.68%) extended into dentin.

**Fig. 4:**
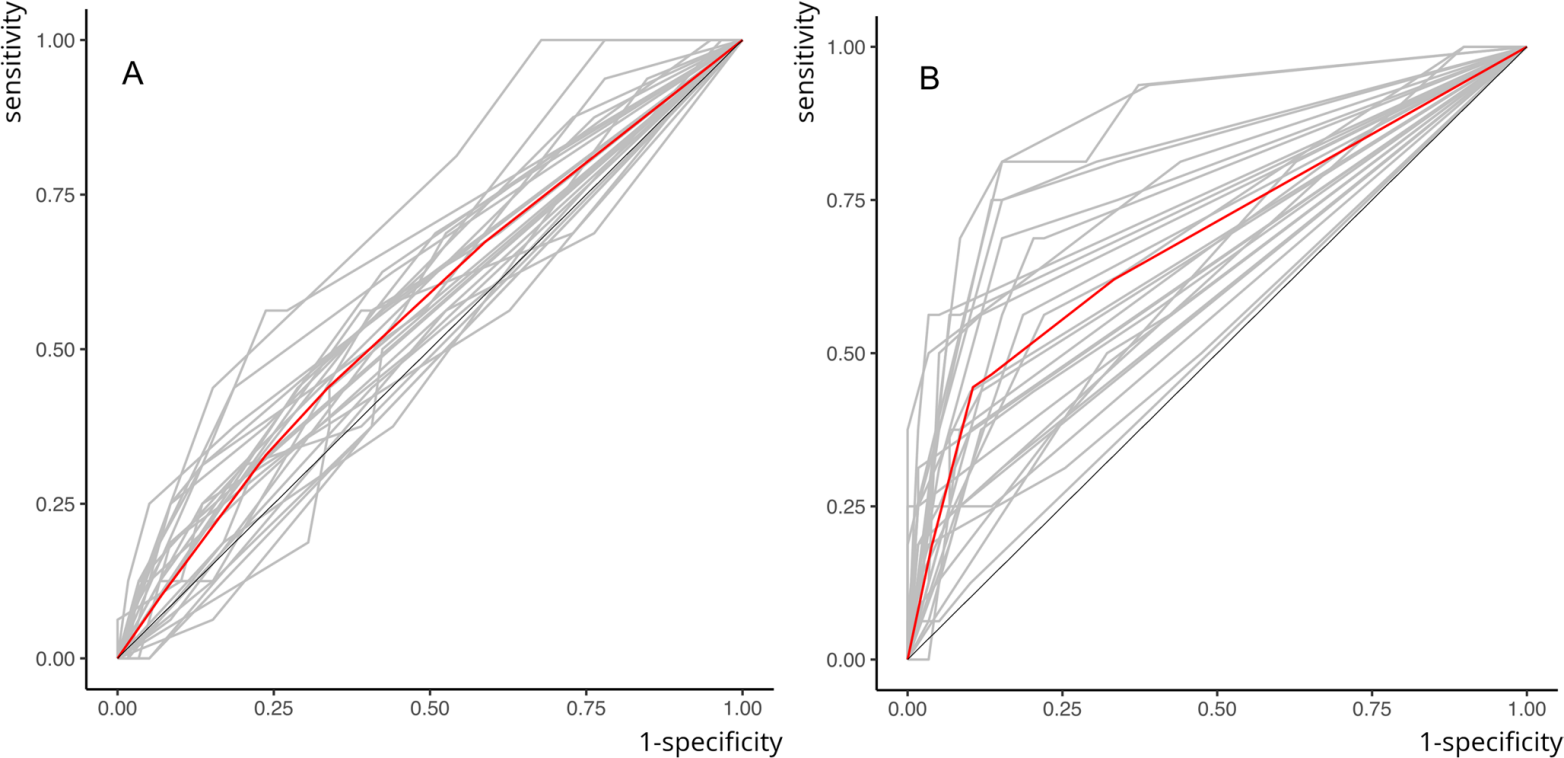
ROC-curves of all 27 readers (gray lines) and a pooled curve over all readers (red curve) for eBWR (**A**) versus iBWR (**B**) for lesions reaching into dentine. Here, iBWR clearly outperforms eBWR

iBWR demonstrated significantly higher AUC (range: 0.513 to 0.875, mean: 0.684 versus 0.475 to 0.723, mean: 0.563 for eBWR), specificity, LR+, PPV, and NPV values for detecting dentin caries at both time points and higher sensitivity at T2 (all *W*s ≥ 86, all *p*s ≤ 0.04, Fig. 4).

**Fig. 5:**
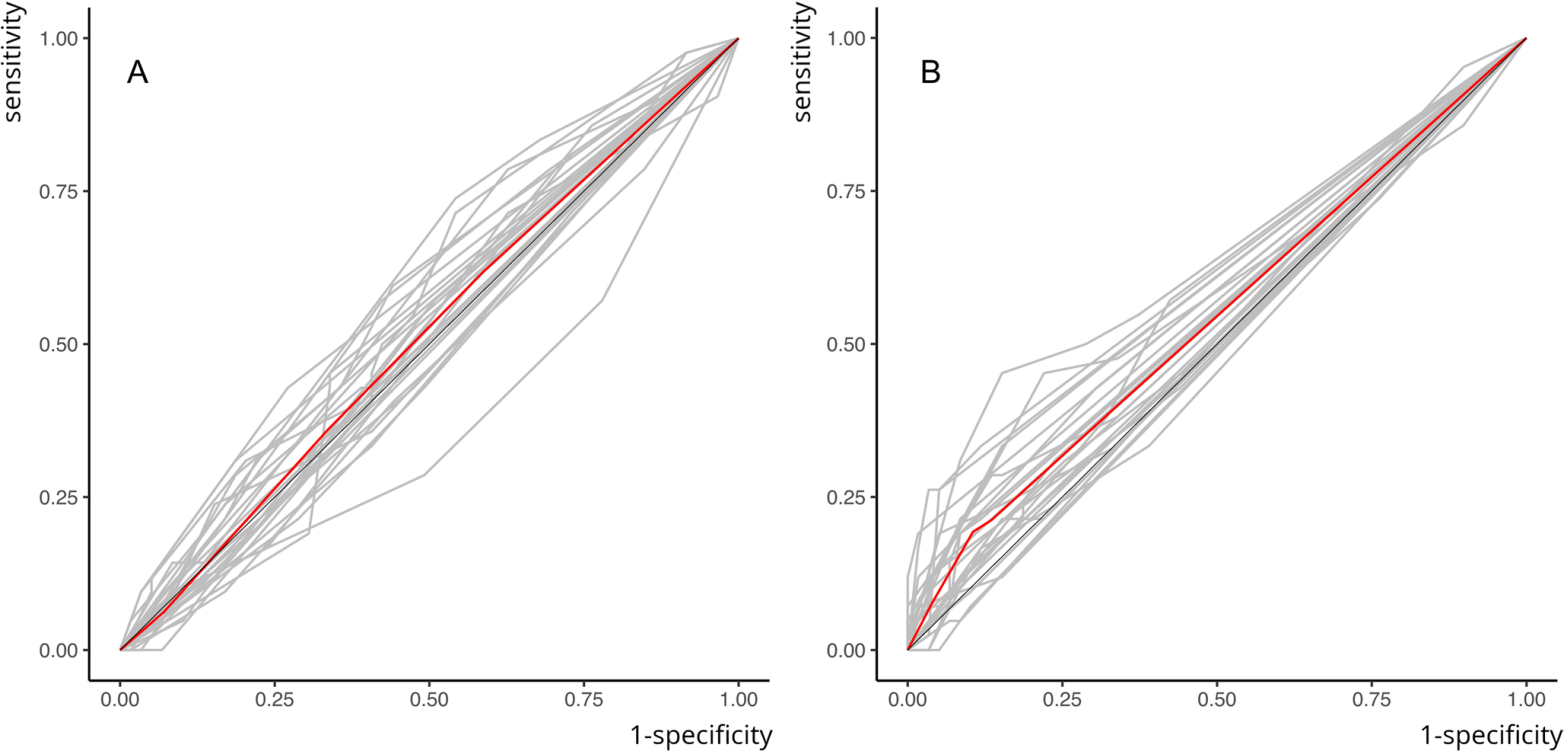
ROC-curves of all 27 readers (gray lines) and a pooled curve over all readers (red curve) for eBWR (**A**) versus iBWR (**B**) for lesions confined to enamel. Clearly, the lines indicate a performance that on average is just above chance

For enamel caries detection, iBWR outperformed eBWR in specificity, PPV, and LR + at both time points (all *W*s $\:\ge\:$ 110, all *p*s $\:\le\:$ 0.03, Fig. 5). AUC ranged between 0.481 and 0.633 (mean: 0.541) for the former, versus 0.377 to 0.606 (mean: 0.518) for the latter technology. However, eBWR demonstrated significantly higher sensitivity (mean ± standard deviation eBWR: 0.564 ± 0.23 vs. 0.268 ± 0.14, *p* < 0.01) at T1. No significant differences were observed between iBWR and eBWR for detecting enamel caries of AUC, NPV, and LR− (all Ws ≥ 64, all *p*s ≥ 0.06). Two-factorial analysis of variance revealed significant influence of lesion depth (*p* < 0.001) and the combination of lesion depth and method (*p* < 0.01) on sensitivity. The imaging method alone did not show a significant influence on sensitivity.

## Discussion

It is a well-known fact that diagnostic accuracy of bitewing radiography for the detection of minute 1 st stage carious lesions is low [26–28]. Despite that fact, these radiographs nevertheless prevail to be acquired in multitude in the clinical world. This is comprehensible, since intraoral BWR, despite their questionable performance for early-stage lesions, represent the state-of-the-art for general inter-proximal caries detection in patients [29]. Not only from a work-process perspective, it would be beneficial to acquire these radiographs without a rather complex placement of an intraoral image receptor in the patients’ oral cavity. Patients would certainly also favor such an option if it were proven equivalent to the state-of-the art. Particulary patients with strong gag reflex or narrow anatomical conditions would benefit from extraoral imaging.

Here extraoral BWR comes into play. Although some authors question the term “extraoral bitewing radiography” per se [30], various panoramic radiography manufacturers now implement programs using this term. This study investigated the diagnostic accuracy of iBWR versus eBWR in detection of real carious lesions in an ex vivo scenario. To remedy the well-known inter-observer variance in radiological diagnosis [11, 12], a multi-observer design was employed. 27 dentists rated the radiographs on a five-point Likert scale. One important difference is the resulting image size, which due to its’ acquisition in a panoramic machine with the line detector has more pixel compared to iBWR (Fig. 2). We used a 1:1 pixel display which should be applied for medical imagery not to waist image information. However, owing to this fact the displayed size of the assessment regions (the tooth crowns) is smaller in eBWR as there is quite a lot anatomy (i.e. the alveolar ridge) included above and below the teeth. Yet this also reflect the clinical situation. How this influences diagnostic accuracy cannot be concluded from our study.

Our results indicate that both intraoral as well as extraoral BWR proved an overall low performance in the detection of interproximal carious lesions. Yet intraoral BWR (AUC-values of ca. 0.58) slightly outperformed their extraoral counterparts (AUC-values of ca. 0.54). Particularly, specificity was higher for iBWR, meaning that it is more likely on them to detect sound interproximal surfaces. In a clinical context, this would reduce the number of unnecessary interventions. Interestingly, for enamel caries the performance difference was even more pronounced. On the other hand, in the first observation round we observed a higher sensitivity for enamel lesions in eBWR. We cannot really explain this finding. This would yield more truly detected enamel lesions. As unnecessary interventions particularly for enamel caries should be avoided, we feel that the risk for false positive “detections” in a patient setting would be less favorable. As AZ-values reflect the performance of a diagnostic system by iteratively shifting the trade-off of between sensitivity and specificity according to the observers’ confidence levels, altogether iBWR outperformed eBWR. This may by partly explained by the spatial resolution differences of the systems. While intraoral sensor-based digital radiography may offer a spatial resolution of between 6 lp/mm and 15 lp/mm [31], the values for digital panoramic radiography range roughly between 2 lp/mm and 3 lp/mm [32]. Abu El-Ela and colleagues, in their study using artificially induced acid lesions, observed no difference between eBWR and iBWR [10]. Although they used the same panoramic device for the eBWR, this study only had two observers, and no real caries was assessed. The authors observed strong interobserver agreement [10] while in our study both inter- as well as intra-rater reproducibility were low. This can be also attributed to the fact, that diagnostic accuracy of bitewing radiography for the detection of minute 1 st stage carious lesions is low [26–28]. Hence agreement between observers will also be low because they are very insecure on their judgment. Obviously, the Chance of a lower overall agreement rises with the number of observers involved. Obuchowski in 2004 concluded “From the earliest phase to the final phases of assessment, multiple-observer studies are critical to clinical studies of medical imaging” [33]. It seems likely, that specific training with both modalities would enhance agreement between observers. Here, the focus should lie on initial enamel lesion detection. It would also be interesting to investigate, how a well-trained deep learning system performs for this specific task.

We believe that the relatively small differences in performance of the two modalities could only be detected due to the high observer number involved in our study. Another study comparing eBWR versus iBWR in real patients found higher sensitivity yet a lower specificity for caries detection in the former modality [34]. However, this study suffers from the severe, yet in a patient study unavoidable drawback that the definition of a carious lesion was made by consensus radiographic diagnosis from 5 observers. This lack of a gold standard has been shown to overestimate accuracy and to potentially be misleading [9]. We identified two studies using histological sections for assessment of ground truth [7, 35]. Both studies observed substantially higher AZ-values (> 0.8 [7] and > 0.7 [35]). We believe the low values we observed can be explained by the high prevalence of lesions confined to the enamel and a low proportion extending into dentin. This made the diagnostic task for the observers rather complex. In summary our findings suggest, that intraoral bitewing radiography performs somewhat better for the generally difficult task to detect an initial carious interproximal lesion detection. The small but significant difference raises the question of the extent to which it is clinically relevant. This is particularly true with regard to today’s much less invasive caries therapy. Thus, the question on clinical relevance needs to be addressed in the future by patient outcome-centered studies.

Despite enhanced patient comfort, extraoral bitewing radiography also has some drawbacks. Effective dose has been shown to be higher compared with that imposed by intraoral BWR [36]. In addition, eBWR requires a fancy panoramic radiography device, which is certainly not as globally available as intraoral radiography equipment.

Our study also has limitations. Although we tried very hard to produce a model most closely resembling the natural situation, obviously the natural teeth were placed in plaster models and not real bone. This altered the radiographic appearance at least in the root region of the resulting BWR. It can only be speculated if this had an influence on observer performance and thus resulting accuracy measures. The PMMA-phantom also is not producing a real-world scattering environment. Hence, the absolute accuracy values (sensitivity and specificity) may likely overestimate diagnostic accuracy in a real world scenario. Using real patient radiographs would be a solution here, but with the considerable disadvantage of a missing gold standard [18]. Unfortunately, there is as yet no approach to using both real patient radiographs and at the same time a robust reliable gold standard for caries status. Despite this obvious shortcoming, our study due to its design was able to detect rather small differences in diagnostic performance of the two modalities. Thus subject to the above restrictions we believe that the results are relevant for daily practice. However, it is also clear that future studies should focus on patient outcome to provide more insight on the clinical implications of the small differences.

In conclusion, our results indicate a slightly yet significantly higher overall accuracy in detecting carious lesions for intraoral BWR. The study also confirms that diagnostic accuracy for minute enamel lesions is, at best slightly higher than random guess. For dentin lesions, both intraoral and extraoral BWR showed equal performance except of the specificity, which was higher for iBWR. Altogether our results indicate that the gold standard for radiographic interproximal caries remains to be intraoral bitewing radiography, however the patient-centered effect caused by the performance difference needs to be still investigated.

## Data Availability

The scientific raw data of this study can be provided as an excel-file upon reasonable request
